# Supplementary material for: Pattern and predictor of hookworm re-infection among schoolchildren in three districts of Amhara Region, northwest Ethiopia
Source: PLoS One. 2024 May 28;19(5):e0303502. doi: 10.1371/journal.pone.0303502 (PMC11132481; doi:10.1371/journal.pone.0303502)
Supplement: Attachment — Submitted filename: PONE-D-23-41533 edited.pdf [file pone.0303502.s001.pdf]

PLOS ONE

Pattern and predictor of hookworm re-infection among schoolchildren in three districts of Amhara Region, northwest Ethiopia

--Manuscript Draft--

**Manuscript Number:**	PONE-D-23-41533
**Article Type:**	Research Article
**Full Title:**	Pattern and predictor of hookworm re-infection among schoolchildren in three districts of Amhara Region, northwest Ethiopia
**Short Title:**	Pattern and predictor of hookworm re-infection among schoolchildren
**Corresponding Author:**	Tadesse Hailu Jember<br>Bahir Dar University College of Medical and Health Sciences<br>BAhir Dar, ETHIOPIA
**Keywords:**	Hookworm, Re-infection Rate, Predictors, Albendazole, Ethiopia
**Abstract:**	Background: Despite integrated prevention and control measures, the prevalence of hookworm is still high in Ethiopia. The re-infection rates and predictors are poorly addressed. Therefore, this study aimed to determine the patterns of hookworm re-infection rates and predictors among schoolchildren in northwest Ethiopia.<br>Methods: A follow-up study was conducted among 86 schoolchildren from May to December 2022. Data on predictors was collected using a questionnaire. Stool samples were collected and processed via Kato-Katz, McMaster, and spontaneous tube sedimentation techniques. All hookworm-infected children were treated with albendazole and followed for six months. The re-infection rates of hookworm were checked in the 4th and 6th months. Data was entered into Epi-data version 3.1 and analysed using SPSS version 25. Descriptive statistics were used to compute the re-infection rate. The associations of predictors with hookworm re-infection rates were calculated by logistic regression. Variables with a p-value < 0.05 were considered statistically significant.<br>Results: Of the 86, 81 schoolchildren completed the study. The prevalence of hookworm re-infection in the 4th and 6th months was 23.5% and 33.3%, respectively. Living with family members greater than five (p =.017), poor utilization of latrine (p =.008), infrequent shoe wear (p =.039), and participating in irrigation (p =.020) were the predictors significantly associated with hookworm re-infections.<br>Conclusions: The re-infection rate was high during the 4th and 6th months. Participating in irrigation, infrequent shoe wear, and poor latrine utilization were predictors of hookworm re-infection. Therefore, mass drug administration, regular shoe wearing, and health education should be advocated.
**Order of Authors:**	Tadesse Hailu Jember
	Shegaw Belay
	Getaneh Alemu
**Additional Information:**	
**Question**	**Response**
**Financial Disclosure**<br><br>Enter a financial disclosure statement that describes the sources of funding for the work included in this submission. Review the submission guidelines for detailed requirements. View published research articles from *PLOS ONE* for specific examples.	The author(s) received no specific funding for this work.

Powered by Editorial Manager® and ProduXion Manager® from Aries Systems Corporation

This statement is required for submission and **will appear in the published article** if the submission is accepted. Please make sure it is accurate.<br><br>**Funded studies**<br>Enter a statement with the following details:<br>• Initials of the authors who received each award<br>• Grant numbers awarded to each author<br>• The full name of each funder<br>• URL of each funder website<br>• Did the sponsors or funders play any role in the study design, data collection and analysis, decision to publish, or preparation of the manuscript?<br><br>Did you receive funding for this work?	
**Competing Interests**<br><br>Use the instructions below to enter a competing interest statement for this submission. On behalf of all authors, disclose any competing interests that could be perceived to bias this work—acknowledging all financial support and any other relevant financial or non-financial competing interests.<br><br>This statement is required for submission and **will appear in the published article** if the submission is accepted. Please make sure it is accurate and that any funding sources listed in your Funding Information later in the submission form are also declared in your Financial Disclosure statement.<br><br>View published research articles from *PLOS ONE* for specific examples.	The authors have declared that no competing interests exist.

Powered by Editorial Manager® and ProduXion Manager® from Aries Systems Corporation

**NO authors have competing interests**<br><br>Enter: *The authors have declared that no competing interests exist.*<br><br>**Authors with competing interests**<br><br>Enter competing interest details beginning with this statement:<br><br>*I have read the journal's policy and the authors of this manuscript have the following competing interests: [insert competing interests here]*<br><br>* typeset	
**Ethics Statement**<br><br>Enter an ethics statement for this submission. This statement is required if the study involved:<br><br>• Human participants<br>• Human specimens or tissue<br>• Vertebrate animals or cephalopods<br>• Vertebrate embryos or tissues<br>• Field research<br><br>Write "N/A" if the submission does not require an ethics statement.<br><br>General guidance is provided below. Consult the submission guidelines for detailed instructions. **Make sure that all information entered here is included in the Methods section of the manuscript.**	N/A

**Format for specific study types**<br><br>**Human Subject Research (involving human participants and/or tissue)**<br>• Give the name of the institutional review board or ethics committee that approved the study<br>• Include the approval number and/or a statement indicating approval of this research<br>• Indicate the form of consent obtained (written/oral) or the reason that consent was not obtained (e.g. the data were analyzed anonymously)<br><br>**Animal Research (involving vertebrate animals, embryos or tissues)**<br>• Provide the name of the Institutional Animal Care and Use Committee (IACUC) or other relevant ethics board that reviewed the study protocol, and indicate whether they approved this research or granted a formal waiver of ethical approval<br>• Include an approval number if one was obtained<br>• If the study involved *non-human primates*, add *additional details* about animal welfare and steps taken to ameliorate suffering<br>• If anesthesia, euthanasia, or any kind of animal sacrifice is part of the study, include briefly which substances and/or methods were applied<br><br>**Field Research**<br><br>Include the following details if this study involves the collection of plant, animal, or other materials from a natural setting:<br>• Field permit number<br>• Name of the institution or relevant body that granted permission	
**Data Availability**<br><br>Authors are required to make all data underlying the findings described fully available, without restriction, and from the time of publication. PLOS allows rare exceptions to address legal and ethical concerns. See the PLOS Data Policy and FAQ for detailed information.	Yes - all data are fully available without restriction

A Data Availability Statement describing where the data can be found is required at submission. Your answers to this question constitute the Data Availability Statement and **will be published in the article**, if accepted.<br><br>**Important:** Stating 'data available on request from the author' is not sufficient. If your data are only available upon request, select 'No' for the first question and explain your exceptional situation in the text box.<br><br>Do the authors confirm that all data underlying the findings described in their manuscript are fully available without restriction?	
**Describe where the data may be found in full sentences. If you are copying our sample text, replace any instances of XXX with the appropriate details.**<br><br>• If the data are **held or will be held in a public repository**, include URLs, accession numbers or DOIs. If this information will only be available after acceptance, indicate this by ticking the box below. For example: *All XXX files are available from the XXX database (accession number(s) XXX, XXX.).*<br>• If the data are all contained **within the manuscript and/or Supporting Information files**, enter the following: *All relevant data are within the manuscript and its Supporting Information files.*<br>• If neither of these applies but you are able to provide **details of access elsewhere**, with or without limitations, please do so. For example:<br><br>*Data cannot be shared publicly because of [XXX]. Data are available from the XXX Institutional Data Access / Ethics Committee (contact via XXX) for researchers who meet the criteria for access to confidential data.*<br><br>*The data underlying the results presented in the study are available from (include the name of the third party*	All relevant data are within the manuscript and its Supporting Information files.

and contact information or URL).<br>• This text is appropriate if the data are owned by a third party and authors do not have permission to share the data.<br><br>* typeset	
Additional data availability information:	Tick here if the URLs/accession numbers/DOIs will be available only after acceptance of the manuscript for publication so that we can ensure their inclusion before publication.

Manuscript

Click here to access/download;Manuscript;Hookworm re-infection final best.docx

Pattern and predictor of hookworm re-infection among schoolchildren in three districts of Amhara Region, northwest Ethiopia

Shegaw Belay[1], Getaneh Alemu[2], Tadesse Hailu[2*]

[1]Amhara National Regional State Health Bureau, Bahir Dar Health Science College, Bahir Dar, Ethiopia;

[2]Departments of Medical Laboratory Science, College of Medicine and Health Sciences, Bahir Dar University, Ethiopia;

***Corresponding author**: Tadesse Hailu (PhD) E.mail:tadessehailu89@gmail.com

Abstract

Background: Despite integrated prevention and control measures, the prevalence of hookworm is still high in Ethiopia. The re-infection rates and predictors are poorly addressed. Therefore, this study aimed to determine the patterns of hookworm re-infection rates and predictors among schoolchildren in northwest Ethiopia.

Methods: A prospective follow-up study was conducted among 86 schoolchildren from May to December 2022. Data on predictors was collected using a questionnaire. Stool samples were collected and processed via Kato-Katz, McMaster, and spontaneous tube sedimentation techniques. All hookworm-infected children were treated with albendazole and followed for six months. The re-infection rates of hookworm were checked in the 4th and 6th months. Data was entered into Epi-data version 3.1 and analysed using SPSS version 25. Descriptive statistics were used to compute the re-infection rate. The associations of predictors with hookworm re-infection rates were calculated by logistic regression. Variables with a *p-value* < 0.05 were considered statistically significant.

Results: Of the 86, 81 schoolchildren completed the study. The prevalence of hookworm re-infection in the 4th and 6th months was 23.5% and 33.3%, respectively. Living with family members greater than five (p =.017), poor utilization of latrine (p =.008), infrequent shoe wear (p =.039), and participating in irrigation (p =.020) were the predictors significantly associated with hookworm re-infections.

Conclusions: The re-infection rate was high during the [illegible] and 6th months. Participating in irrigation, infrequent shoe wear, and poor latrine utilization were predictors of hookworm re-infection. Therefore, mass drug administration, regular shoe wearing, and health education should be advocated.

Keywords: Hookworm, Re-infection Rate, Predictors, Albendazole, Ethiopia

Introduction

Hookworms is one of the soil-transmitted helminths (STHs) that dwell in the small intestine of human beings. The two main spp. of hookworm that infect humans are *Ancylostoma duodenale* and *Necator americanus*. They mainly affect the poorest and most deprived communities with poor nutrition, inadequate sanitation, overcrowding, and barefoot (1-3).

Hookworm species were responsible for more than 4 million disability-adjusted life years (DALYs), and more than 500 million people were infected in tropical countries (4). Of these, nearly 120 million live in sub-Saharan Africa (5).

Ethiopia had the third highest sub-Saharan African hookworm infection burden (6). Despite control measures implemented in the country, the prevalence of hookworm among children remains high. The problem is severe in poor and rural communities (7, 8). Previous, reports in Ethiopia indicated that the prevalence of hookworm was high. For instance, the prevalence of hookworm spp. was 15.3% in southern Ethiopia, 48.2% in west Gojjam, 46.9% in Durbete town, 41.3% in Sebatamit, and (64.2–87.7%) in rural Bahir Dar, Amhara Region (8-11).

Hookworm re-infection rates reveal continuous exposure to the sources of infections in endemic communities (12, 13). Substantial re-infection after complete deworming of hookworm infection is a common problem (14, 15). The re-infection can be occur as early as two months after treatment in areas with high transmission settings (16). For example, a study conducted in Malaysia showed that 51.8% of the treated population becomes re-infected by hookworm 6 month, after deworming. Similarly, 5.1% of schoolchildren were re-infected by hookworm 6 months post treatment in Yunnan, People's Republic of China (16).

Several factors including socio-demographic, socioeconomic, behavioral, and environmental factors could be the predictors of hookworm re-infection (17). Hence, the prevention of hookworm

requires an integrated prevention chemotheraapy, implementing improved water, sanitation, and hygiene (WASH), and a community-based health education strategy in endemic areas (18). Although our study area is endemic for hookworm infection, the re-infection rates of hookworm and asscociated factors to the re-infection are not yet well determined. Therefore, this study aimed to determine the re-infection rate, and predictor of hookworm re-infection among schoolchildren in three endemic districts of Amhara Region, northwest Ethiopia.

Materials and methods

Study design, period, and area

A school-based prospective follow-up study was conducted from May to December 2022 to determines the re-infection rates of hookworm and associated factors in three selected districts of Amhara Region, Northwest Ethiopia. A total of 403 schoolchildren were examined to check single infection of hookworm. All hookworm positive schoolchildren in selected primary schools, whose ages ranged from 6 to 14 years, lived for the last six months in the study area prior to data collection, and whose parents/guardians gave written consent for their children to participate and take albendazole were included in the study. Lastly, schoolchild who gave assent and a stool sample in the 4th and 6th months post treatment were included. Those schoolchildren who received any anti-helminthic drug within the last three months prior to data collection were excluded from the study.

Sample size and sampling technique

Three districts namely: North Mecha, Bahir Dar City administration and Bahir Dar zuria districts were selected as a study site. One school from each district was randomly selected by a lottery method. The sample size was proportionally allocated to each school by considering the total number of students. Systematic random sampling technique was used to select participants in each school by using the class roster as a sampling frame. Stool sample was collected from each

schoolchildren and those children positive to hookworm infection were treated with albendazole. A total of 86 albendazole treated and cured from hookworm infection schoolchildren who provide assent to participate in the follow-up study were included (Fig 1).

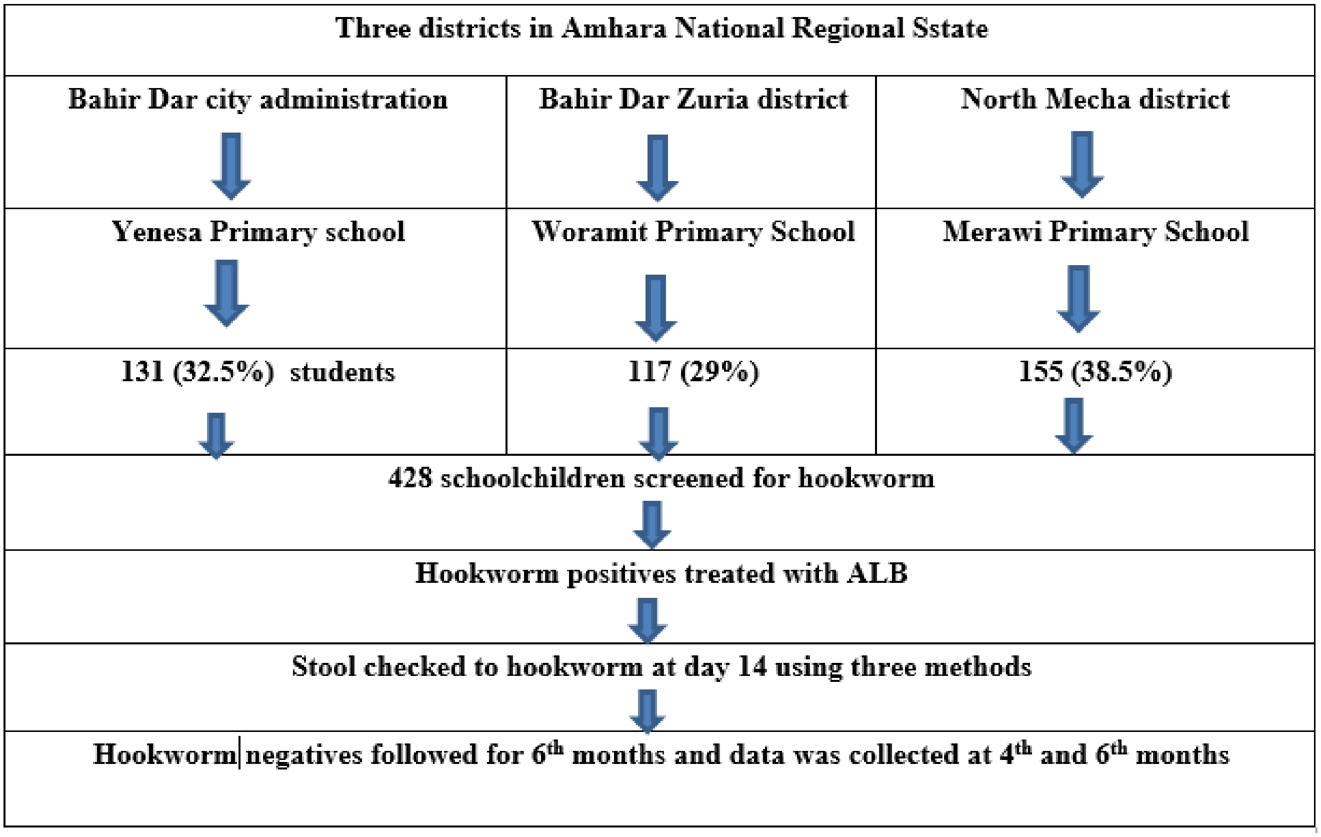

Figure 1: Sampling frame that shows how study sites, schools and schoolchildren were selected, 2022

Data collection methods

A structured questionnaire was used to collect data on socio-demographical and factors associated with hookworm re-infection. The data was collected by trained laboratory professionals. Fresh stool samples were collected using a clean and leak-proof stool container labeled with the participant's unique identification number to each study participant. Stool sample[illegible]llected and transported to laboratory center with in an hour of collection. Each stool sample was processed

and examined following standard operating procedure. Three sensitive diagnostic methods were used for the diagnosis purpose.

Spontaneous tube sedimentation technique (STST): Nearly three grams of fresh stool sample was weighed, homogenized in 10 ml of normal saline solution and mixed well. The mixture was filtered through wire mesh into a 50 ml falcon tube, which was then filled with more saline solution up to 50 ml gauge, plugged and shaken vigorously. The falcon tube was left to stand for 45 minutes to settle. The sediment was taken with Pasteur pipette, put on a microscope slide and examined with 10 x followed by 40 x objectives of a microscope to check for the presence of ova of hookworm (19).

Kato-Katz (KK): About two-three grams of fresh stool sample was pressed through a mesh screen to remove large particles. Around 41.7 mg of stool was sieved and transferred to the template which was put on a slide until the template hole was filled. Then, the template was removed and the stool sample was covered and pressed with cellophane which was previously immersed overnight with Glycerol-malachite green. The KK smears were examined within 30-60 minutes for hookworm spp. (20).

McMaster (MM): Approximately, two grams of fresh stool was suspended in 30 ml of saturated salt (NaCl) solution at room temperature. The suspension was filtered through a mesh and well mixed 10 times by using Pasteur pipette. About 0.15 ml aliquot was added to each side of a MM slide chamber, waited two minutes to settle and examined under a light microscope with 10 x objectives. The fecal egg count of hookworm spp. was calculated by multiplying the fecal egg count by 50 (21).

Re-infection rate assessment

Children that [illegible] positive for hookworm infections during the baseline survey were treated with albendazole and microscopic negative ([illegible]es) after 14 days of post-treatment were re-tested in the 4th and 6th months follow-up survey using the same laboratory techniques.

$$\text{Re-infection rate} = \frac{\text{Number of infected children after treatment}}{\text{Number of negative children at follow-up who were positive at baseline}} \text{ X100}$$

Data quality control

Training was given for both questionnaire-based and laboratory-based data collectors. The questionnaire data was checked daily for completeness by the principal investigator. The amount of stool sample and consistency was checked. Two laboratory personnel examined the slides independently. The result of their observation was recorded separately for later comparison. All discordant results were re-checked. Ten percent of the slides were cross checked by principal inv[illegible]gettor. Generally, To secured the reliability of the finding, quality control was che[illegible]d at pre-analytical, analytical, and post-analytical quality control steps.

Data management and analysis

All data were entered into Epi Info version 3.1 and analyzed with SPSS version 25 software.. Descriptive statistics were used to compute the re-infection rate. The associations of independent variables with the re-infection rate of hookworm were calculated by bivariate logistic regression analysis. All variables with *P-values* < 0.20 in the bivariate analysis were run in a multivariate logistic regression analysis to filter the co-f[illegible]ding effects. Variables with *p-value* < 0.05 were considered statistically significant.

Ethical consideration

Ethical clearance was obtained from the College of Medicine and Health Sciences Institutional Review Board, Bahir Dar University (protocol numbe: 384/2022). Permission [illegible] also obtained

from the Bahir Dar City administration, North Mecha district, and Bahir Dar Zuria district health and education offices. Furthermore, written informed consent from the parent/guardian and assent from schoolchildren was secured. Confidentiality of the collected information and laboratory test results were maintained. Hookworm positive cases were treated with a single-dose albendazole (400 mg). Finally, those who tested positive for any intestinal parasitic infefctions were referred to nearby healthcare facilities for treatment.

Results

Socio-demographic characteristics of study participants

Among the 423 schoolchildren in this study, 403 gave complete data and provided stool samples in the baseline survey with a response rate of 94.2%. Among the 403 participants, 223 (55.3%) were males. The mean age of participants was 10.84 ± 2.04 SD years. The study participants were selected from three districts in Amhara Region: 131 (32.5%) participants from Bahir Dar city administration, 155 (38.5%) participants from North Mecha district, and 117 (29%) participants were selected from Bahir Dar Zuria district (Table 1).

Table 1: Socio-demographic characteristics of schoolchildren participated in the baseline survey attending selected schools in Amhara Region, northwest Ethiopia, from May to December 2022 (N=403)

Variables		Frequency n (%)	IP n (%)
Sex	Male	223 (55.3)	104 (46.6)
	Female	180 (44.7)	64 (35.6)
Age	6-10	156 (38.7)	45 (28.8)
	11-14	247 (61.3)	123 (49.8)
	Rural	308 (76.4)	134 (43.5)

Residence	Urban	95 (23.6)	34 (35.8)
	North Mecha	155 (38.5)	53 (34.2)
Participant districts	Bahir Dar city	131 (32.5)	51 (38.9)
	Bahir Dar Zuria	117 (29.0)	64 (54.7)

The prevalence of hookworm infection among schoolchildren from the baseline data was 115 (27.2%). A total of 86 (33 female and 53 male) participants who were hookworm positive at baseline, treated with albendazole and tested negative after 14 days of post-treatment were followed for 6 months to determine their re-infection rate status. Stool sample was checked in the 4th and 6th months. Five students were losted from follow-up in the 4th month data collection period. Eighty-one (28 female and 53 male) participants gave a stool sample and completed the hookworm re-infection rate follow-up (Fig 2). The majority of participants were males (65.4%), 11-14 age group (82.7%), rural dwellers (75.3%) and North Mecha district (38.3%).

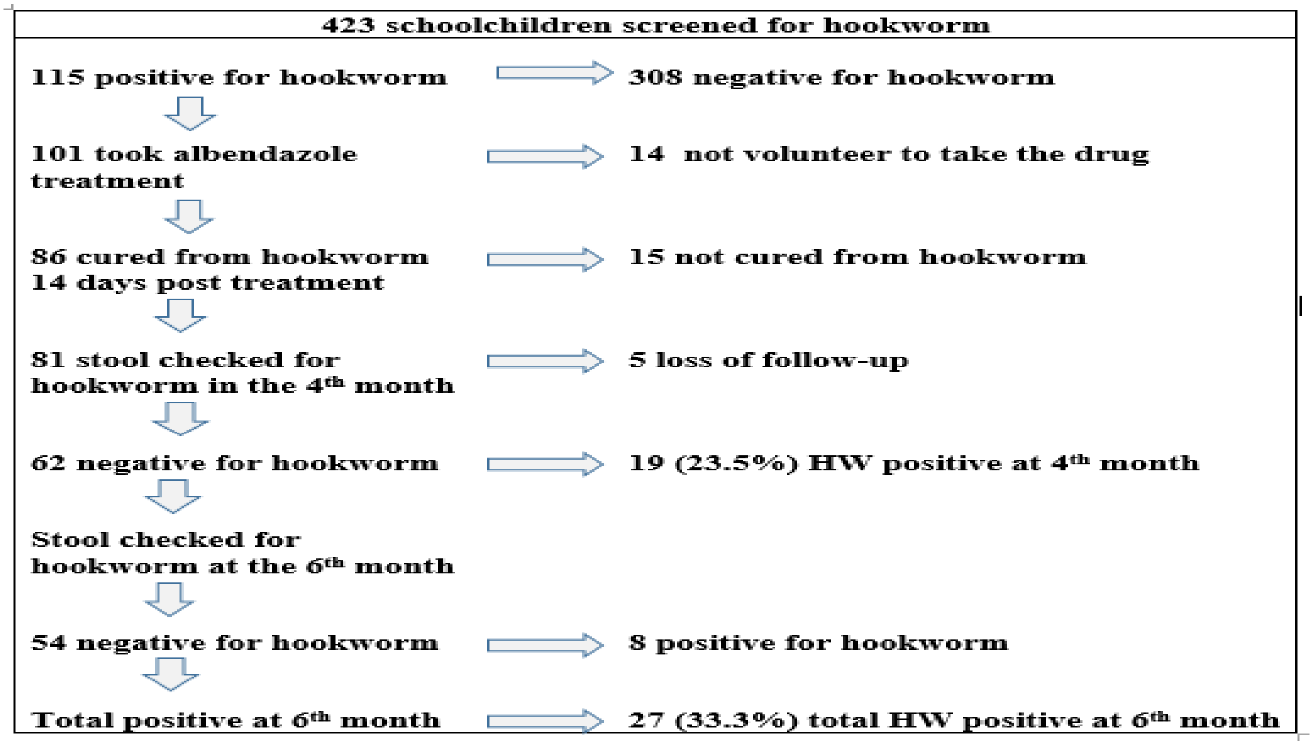

Figure 2: Sampling frame of study participants in the hookworm re-infection study, 2022

Re-infection rate and intensity of hookworm species

The overall re-infection rate of hookworm species among schoolchildren was 23.5% (95% CI 14.8–34.2) in the 4th month and 33.3% (95% CI 23.2-44.7) in the 6th month by the combination of three methods. The re-infection rate of hookworm was 21% by KK, 23.5% by STST, and 23.5% by MM in the 4th month. In the 6th month, the re-infection rate was 29.6% by KK, 33.3% by STST, and 33.3% by MM (Fig 2, Fig 3). In the six months follow-up, 3 (3.7%) *S. mansoni*, 3 (3.7%) *A. lumbricoides*, and 1 (1.2%) *T. trichuria* were also detected.

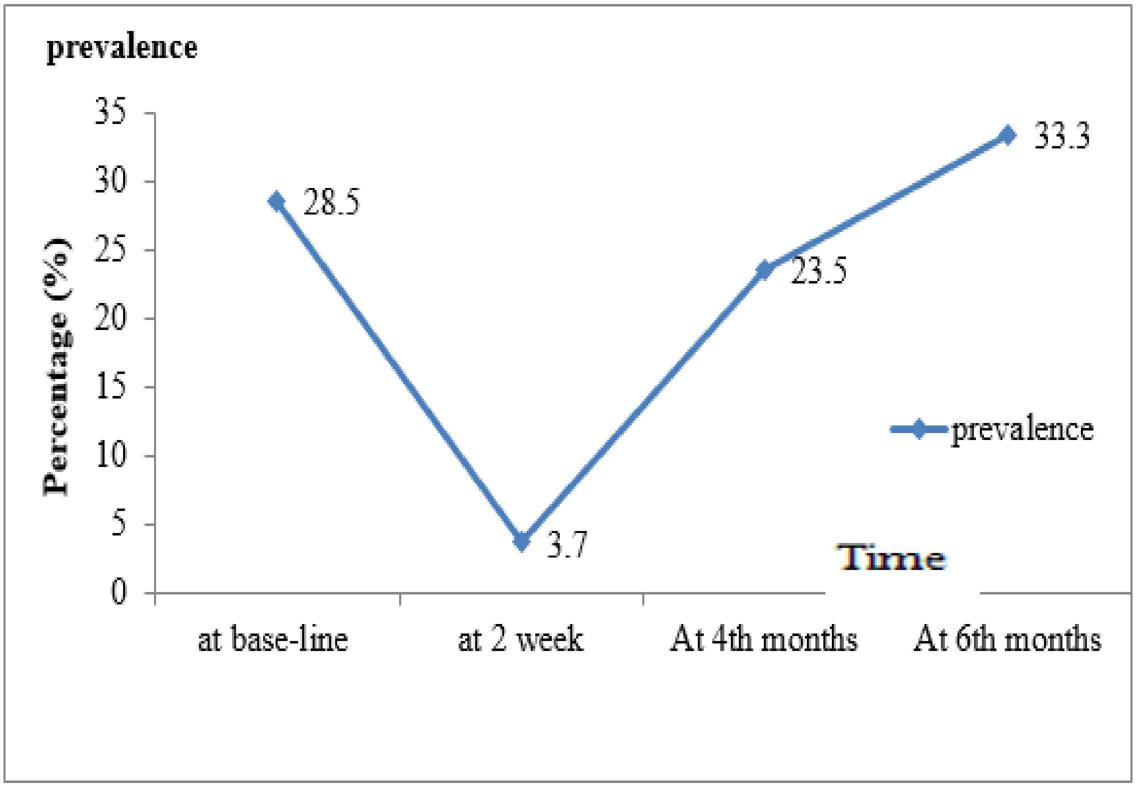

Figure 3: Prevalence of hookworm at baseline, 2 weeks, and 4th and 6th months after deworming by composite reference method

The intensity of infection: The AM intensity of hookworm re-infections in the 4th month was 53.7 EPG and 139.9 EPG by KK and MM methods, respectively. In the 6th month, the AM intensity of infection was increased to 92.6 EPG and 207.1 EPG by the KK and MM methods, respectively. The AM intensity of hookworm re-infections was grouped under “light infection intensity” in the

4th and 6th month follow-up periods based on WHO thresholds for determining infection intensity (Fig 4).

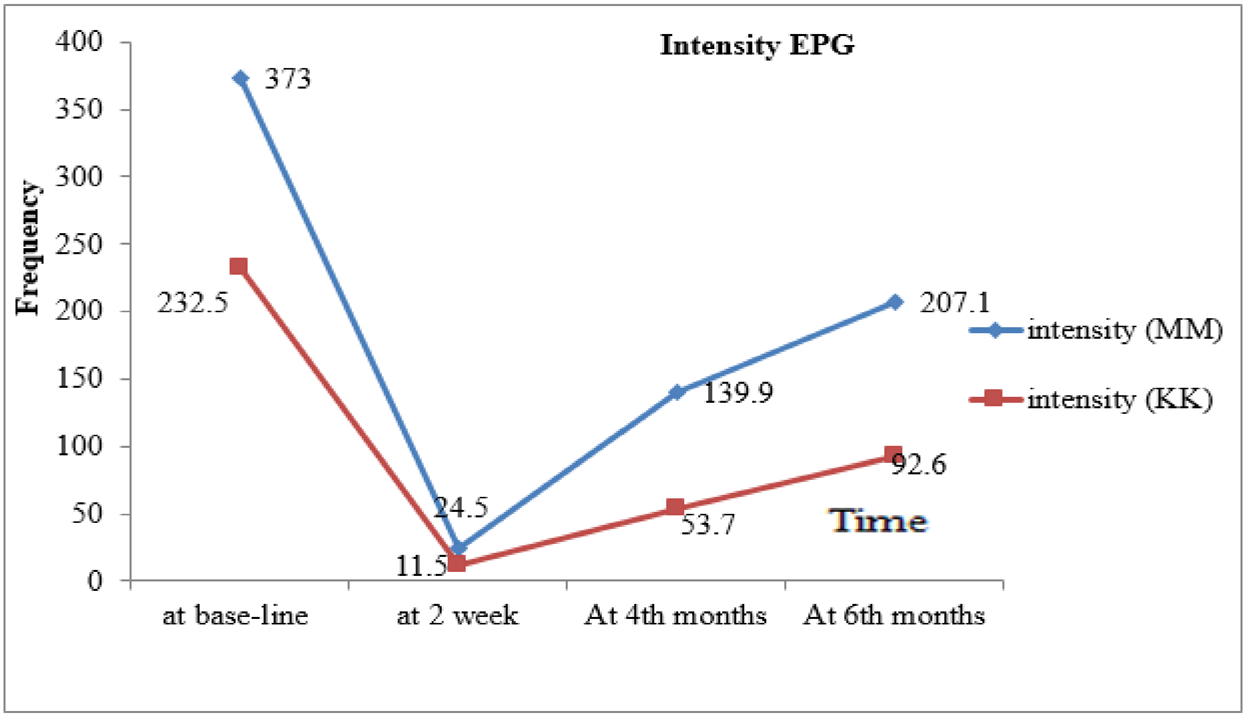

Figure 4: Arithmetic mean intensity of hookworm (EPG) at baseline, two weeks post treatment, $4^{th,}$ and 6th months after deworming by KK and MM diagnostic method, 2022

The re-infection rates of hookworm were 19.4% (95% CI 7.5-37.5), 20.8% (95% CI 7.1-42.2), and 30.8% (95% CI 14.3-51.8) at North Mecha district, Bahir Dar city administration, and Bahir Dar Zuria district, respectively, in the 4th month. In the 6th month, the re-infection rate was 29% (95% CI 14.2-48.0) in the North Mecha district, 42.3% (95% CI 23.4-63.1) in the Bahir Dar Zuria district, and 29.2% (95% CI 12.6-51.1) in the Bahir Dar city administration (Fig 5).

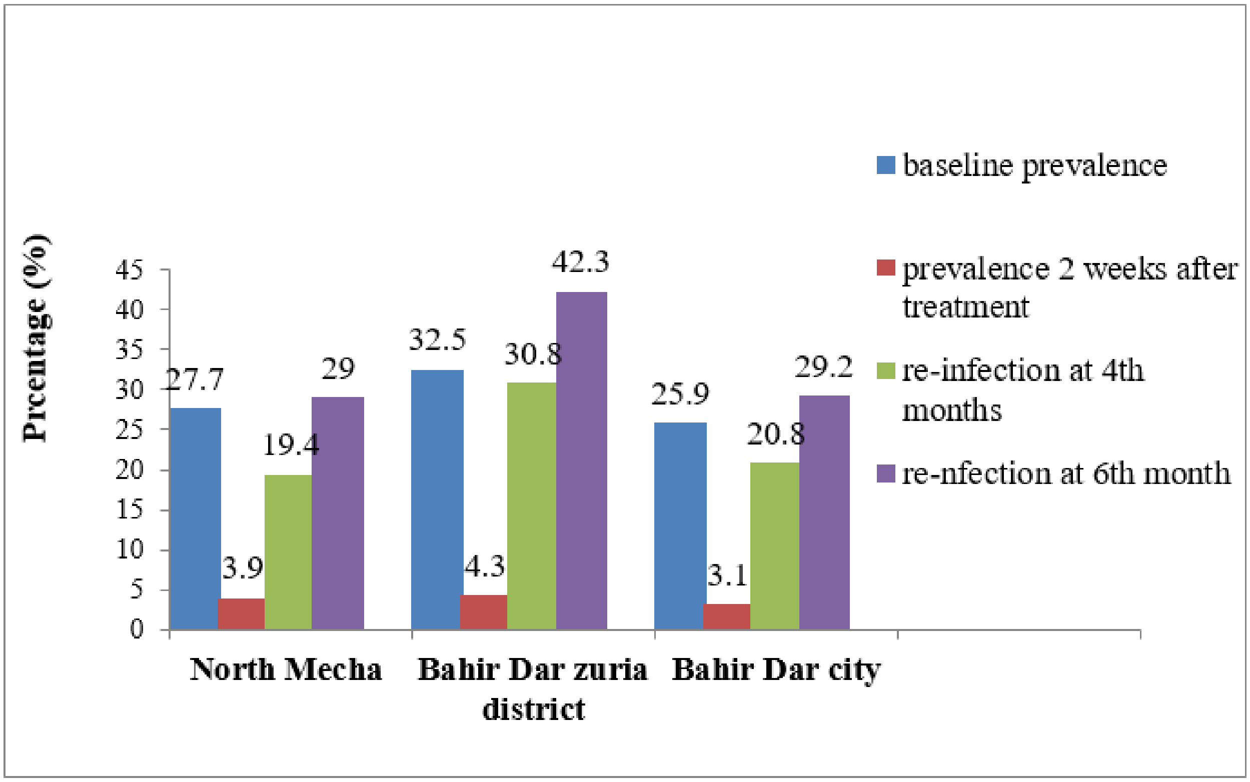

Figure 5: Baseline prevalence, two weeks after treatment, in the 4th, and 6th month re-infection rates of hookworm in Amhara region, northwest Ethiopia

Factors associated with hookworm re-infection

In the bivariate analyses, schoolchildren who practice open defecation (COR=3.186 CI=1.023-9.927, p=.046), poor hand washing practice after using a toilet (COR=2.941 CI=1.020-8.484, p=.046), infrequent shoe wearing (COR=3.877 CI=1.241-12.109), p=.02), participate in irrigation (COR= 3.400 CI=1.091-10.60, p=.035) had higher re-infection rates of hookworm infection than their counter part in the 4th month.

In the multivariate analysis, children who did not wear shoes consistently were 3.4 times more likely to be re-infected by hookworm (AOR =3.359 CI=1.024-11.026, p=.020) in the 4th months. Similarly, schoolchildren who participate in irrigation activity were 3.7 times more likely to be re-infected by hookworm (AOR= 3.704 CI=1.092-12.562, p=.036) than schoolchildren who did not participate in irrigation in the 4th months (Table 2).

Table 2: Bivariate and multivariate analysis of factors associated with hookworm re-infection in the 4th month among schoolchildren in the Amhara Region, northwest Ethiopia, 2022 (n = 81)

**Variables**	**Re-infection rates of hookworm species in 4th month**						
	**Categories**	**Pos. n (%)**	**Neg. n (%)**	**COR (95% CI)**	***p*-value**	**AOR (95% CI)**	***p.* value**
Sex	Female	5 (17.9)	23 (82.1)	1			
	Male	14 (26.4)	39 (73.6)	1.651 (.397-3.574)	.754		
Age	6-10	3 (21.4)	11 (78.6)	1			
	11-14	16 (23.9)	51 (76.1)	1.150 (.285-4.640)	.844		
Family size	=<5	5 (14.7)	29 (85.3)	1			
	>5	14 (19.8)	33 (70.2)	2.461(.790-7.667)	.120	3.010(.874-10.369)	.081
Residence	Urban	2 (10)	18 (90)	1		1	
	Rural	17 (27.9)	44 (72.1)	3.477(.727-16.621)	.118	1.603(.286-8.978)	.591
Latrine utilization	Yes	5 (11)2	33 (88.8)	1			
	No	14 (32.6)	29 (67.4)	3.186 (1.023-9.927)	.046	2.746 (.789-9.555)	.112
Hand washing after the toilet	Yes	9 (16.7)	45 (83.3)	1		1	
	No	10 (37)	17 (63)	2.941 (1.020-8.484)	.046	1.585 (.472-5.326)	.456
Participation in irrigation	No	5 (17.3)	34 (82.7)	1		1	
	Yes	14 (33.3)	28 (66.7)	3.400 (1.091-10.60)	.035	3.704 (1.092-12.56)	.036*
Frequency of shoe wearing	Always	5 (12.2)	36 (87.8)	1		1	
	Sometimes	14 (35)	26 (65)	3.877(1.241-12.109)	.020	3.359 (1.024-11.026)	.046*

***** Pos=positive, Neg=negative, CI=confidence interval, COR=crude odds ratio, AOR=adjusted odds ratio, *=significant association

In the multivariate analysis, schoolchildren who live within more than five family members (AOR= 4.682 95% CI=1.322-16.578, P=.017), have poor latrine utilization (AOR=5.088 95%CI=1.517-17.065, P=.008), do not wear shoe consistently (AOR=3.099 95% CI=1.058-9.083, P=.039), and actively participate in irrigation activities (AOR=4.142 95% CI=1.247-13.753, P=.020) were significantly associated with hookworm re-infection rates than their counterparts in the 6th months (Table 3).

Table 3: Bivariate and multivariate analysis of factors associated with hookworm re-infection in the 6th month among schoolchildren in the Amhara region, northwest Ethiopia (n = 81)

**Variable**	**Re-infection rates of hookworm species in 6th month**						
	**Categories**	**Pos. n (%)**	**Neg. n (%)**	**COR (95%CI)**	***p-value***	**AOR 95% CI**	***p. value***
Family size	<5	7 (20.6)	27 (79.4)	1		1	
	>5	20 (42.6)	27 (57.4)	2.857 (1.038-7.865)	.042	4.682 (1.322-16.578)	.017*
Residence	Urban	3 (15)	17 (85)	1		1	
	Rural	24 (39.3)	37 (60.7)	3.676 (.972-13.905)	.055	1.535 (.328-7.178)	.586
Latrine availability	Yes	6 (16.2)	31 (83.8)	1		1	
	No	21 (47.7)	23 (52.3)	4.717 (1.642-13.55)	.004	1.402 (.263-7.484)	.692

Latrine	Yes	6 (15.8)	32 (84.2)	1		1	
utilization	No	21 (48.8)	22 (51.2)	5.091(1.769-14.65)	.003	5.088 (1.517-17.065)	.008*
Hand wash	Yes	15 (27.8)	39 (72.2)	1		1	
after toilet	No	12 (44.4)	15 (55.6)	2.080 (.793-5.458)	.137	.770 (231-2.564)	.671
Participation	No	8 (20.5)	31 (79.5)	1		1	
in irrigation	Yes	19 (45.2)	23 (54.8)	3.201 (1.194-8.585)	.021	4.142 (1.247-13.753)	.020*
Frequency of	Always	8 (19.5)	33 (80.5)	1		1	
shoe wearing	Sometimes	19 (47.5)	21 (52.5)	3.732 (1.386-10.052)	.009	3.099 (1.058-9.083)	.039*

COR=crude odds ratio, AOR=adjusted odds ratio, *=significant association

DISCUSSION

Hookworm infection is a treatable and preventable disease with high public health importance in endemic countries like Ethiopia. The national control program was designed to eliminate this parasitic disease by 2025 and achieve less than 2% prevalence using a single dose of ALB (400 mg) or MEB (500 mg), WASH, health education, and advocacy (22). Reports showed that re-infections of hookworm occur quickly after treatment and significantly affect the success of preventive chemotherapy (15, 16).

The baseline prevalence of hookworm infection among schoolchildren was 27.2% (95% CI 23.0–31.7) by the composite reference method. This result is in line with the study conducted in South Sumatra, Indonesia (25.9%) (23) and North Gondar (26.2%) (11). However, the current result is lower than the studies in Debre Elias and Sanja districts, Amhara Region (63.2%) (24), and rural communities in Bahir Dar, Amhara Region (46.9%) (10). The variation could be attributed to differences in study participant age (the previous study included adults), parasite endemicity (level

of soil contamination), diagnostic methods used, and health status of participants (Zeleke used five methods, and the study participants were symptomatic adults). On the contrary, the current finding is higher than the study in Central Kenya (1%) (3) and in Bibugn, Amhara Region (7.5%) (25). This variation could be attributed to the diagnostic method (only the KK method was used in Kenya, but three methods were used in the current study), temperature, and altitude (in Bibugn, there is cold weather at 9-24 °C, which is not comfortable for hookworm larvae survival, development, and transmission).

The post-treatment re-infection patterns of hookworm species largely depend on the degree of endemicity within the community and the level of soil contamination. In the current endemic districts of the Amhara region, follow-ups in the 4th and 6th months after the administration of single-dose 400 mg ALB revealed that re-infection with hookworm was rapid in terms of prevalence. However, the intensity was light. Indeed, the re-infection rate of hookworm reached 23.5% and 33.3% in the 4th and 6th months after treatment, respectively. This increase hookworm re-infection in the 4th and 6th months of follow-up was probably attributable to the convenient soil temperatures and humidity in the summer season (June–October), the soil type in the study area (clay and sandy), and the level of soil contamination in the area. This condition favors the survival, development, and transmission of hookworm larvae. Furthermore, the school was closed in the summer season, and children spent most of their time in the field (they defecate in an open field at a farm, which is conducive to parasite survival and distribution). Moreover, there is also a habit of walking barefoot in the agricultural area in the summer; this could be the cause of the high hookworm re-infection rate during the study period.

The re-infection rate of hookworm in the 4th month among the SC was 23.5% (95% CI 14.8–34.2%), which consistent with previous studies in Tanzania (25.0%) (26) and Debre Elias District, Amhara Region (21.4%) (27). However, this result is higher than that of the Chinese study (2%)

(16). The difference might be due to seasonal differences. In China, the study was conducted from December–March, which is a cold and dry season that is unsuitable for the development and survival of hookworm larvae), the baseline prevalence, the level of soil contamination, and the dose of ALB (the previous study used a triple dose). This result is also higher than the study in Chencha district, southern Ethiopia (1%) (28). The difference might be due to follow-up time, STH species difference, and diagnostic method differences. In Chencha, re-infection was assessed at the 3rd month and used only the KK method, but there is one extra month in this study that allows more time for schoolchildren to become newly re-infected and for surviving infections to re-establish and release eggs.

In the 6th month, the re-infection rate of hookworm was 33.3% (95% CI 23.2–44.7). This result is in line with the study in Myanmar (28.07%) (17). However, this result was lower than the study in Malaysia (51.8%) (29) and systematic review and meta-analysis (55%) (30). The difference could be due to study time variation, during which many intervention activities have been done in the country. This decreases hookworm prevalence through implementing the mass drug administration (MDA) program among school-age children, creating an open defecation-free environment, and house-to-house education by health extension workers. On the contrary, this result is higher than the study in Yunnan, China (5.1%) (16). The difference might be due to the study season difference; the previous study was done in the winter season (December–March), which was a cold and dry condition known to be unsuitable for the development and survival of hookworm larvae. Similarly, the current finding is higher than the study in northwest Ethiopia, at 7.5% after one year (31). The difference could be due to the diagnostic methods and species differences in Zeleke's study, in which direct wet mount and KK methods were used.

In both the 4th and 6th months, there was a slightly higher rate of re-infection in the Bahir Dar Zuria district than in the North Mecha district and Bahir Dar city administration in the current study. In

the 4th month, Bahir Dar Zuria district participants were nearly 1.7 and 1.9 times more likely to be re-infected by hookworm than participants from Bahir Dar city administration and north Mecha district, respectively. At the same time, Bahir Dar Zuria district participants were 1.8 times more likely to be re-infected than Bahir Dar city administration and north Mecha district participants in the 6th month. But the difference was not statistically significant. A slight increase in Bahir Dar Zuria might be due to the initial prevalence and level of environmental sanitation in the school compound, where there was open defecation. During the visits to the study areas, we observed that the school's hygiene was poor; the class floor was covered in mud and dust, many of the students were barefooted, and there was no water for drinking or hand washing after using the toilet in the school compound.

The present study also assessed factors for hookworm re-infection and revealed that the frequency of shoe wearing was significantly associated with an increased re-infection rate of hookworm. Study participants who do not always wear shoes were 3.4 times more likely to be re-infected by hookworm in the 4th month than those who always wear shoes. This finding agrees with study findings in Kenya (32), Shewa, Ethiopia (33), and western Ethiopia (34). This is because wearing shoes minimizes hookworm transmission by providing a physical barrier and preventing larval penetration through the exposed skin of the feet, thus reducing the risk of infection. Similarly, participants who participated in farming were 3.7 times more likely to be re-infected by hookworm than those who did not participate in agriculture. This result agrees with the study in Timor-Leste (35). The reason could be due to the open defecation behavior of people; most adults deposit their faces in the agricultural fields when performing their activity (an agricultural field is ideal for parasite survival and distribution).

In the 6th month, living with more than five family members and poor utilization of the latrine emerged as risk factors for hookworm re-infection. Study participants with a family size greater

than five were 4.7 times more likely to be re-infected by hookworm than individuals with five family members or fewer. This finding is consistent with the studies in Kenya (32) and western Ethiopia (34). The reason could be the difficulty of buying shoes for the entire family as the family size increases, and the children will be barefooted; this increases hookworm re-infection, and also, if one family member is infected, the transmission will be probable. Similarly, children who defecated in the open field were five times more likely to be re-infected with hookworm than those who used latrines. This finding agrees with the study conducted in Chencha, southern Ethiopia (28). This is because latrine use minimizes hookworm transmission from open defecation, which is conducive to parasite distribution.

Conclusions

The re-infection rate of hookworm was high during the 4th and 6th months after treatment. The intensity of infection during the 4th and 6th month's detection was light. Participating in irrigation and irregular shoe wear were predictors of hookworm re-infection in the 4th month. In addition, participating in irrigation, irregular shoe wear, poor latrine utilization, and livings with large family sizes were also risk factors for hookworm re-infection in the 6th month. Therefore, MDA, regular shoes-wearing and health education on behavioral change interventions to should be strongly advocated to reduce the risk of hookworm re-infections.

Acknowledgements

We thank Bahir Dar University, College of Medicine and Health Sciences, School of Health Sciences, Department of Medical Laboratory Science for ethical approval of the study; study participants for volunteer participation in the study; Amhara National Regional Health Bureau for partial funding

Abbreviations and Acronyms

AM-Artimetic mean, DALY- Disability-Adjusted Life-Years, KK-Kato-Katz, MM- McMaster, PSAC- preschool-age children, SAC-school-age children, STH-soil-transmitted helminth, STST-spontaneous tube sedimentation technique, and WHO-World Health organization.

Declarations

Ethics approval and consent to participate: Ethical clearance was obtained from the College of Medicine and Health Sciences ethical review committee, Bahir Dar University (Ref.Nº:384/2022). A permission letter was obtained from Amhara Public Health Institute (Ref.Nº:03/1334). Furthermore, written informed consent from the parents and assent from each study participant were obtained prior to data collection. All methods were carried out in accordance with relevant guidelines and regulations (Declaration of Helsinki). Confidentiality of the collected information and laboratory test results were maintained. Participants positive for hookworm were treated with single dose ALB. Any intestinal parasitosis other than hookworm were communicated to nearby health centers for treatment.

Concept for publication

NA

Availability of data and materials

All data generated or analyzed during this study are included in this published article.

Competing interests: The authors declare that they have no competing interests.

Funding: Amhara National Regional State Health Bureau cover partial funding.

Authors' Contributions

SB-Participated in the study design, literature search, data collection, investigation, data analysis and interpretation as well as writing original draft paper. **GA-** Participated in the study design, methodology validation, data interpretation, data curation and writing review and editing the final

paper. **TH-** Participated in the study design, methodology validation, data interpretation, data curation and editing the final paper.

References

1. WHO. Accelerating work to overcome the global impact of neglected tropical diseases: a roadmap for implementation. World Health Organization; 2012.
2. Asfaw M, Zerdo Z, Churko C, Seife F, Yihune M, Chisha Y, et al. Preventive chemotherapy coverage against Soil-transmitted helminthiases among school age children in vertical versus integrated treatment approaches: Implications from coverage validation survey in Ethiopia. MedRxiv. 2020.
3. Sartorius B, Cano J, Simpson H, Tusting LS, Marczak LB, Miller-Petrie MK, et al. Prevalence and intensity of soil-transmitted helminth infections of children in sub-Saharan Africa, 2000–18: a geospatial analysis. The Lancet Global Health. 2021;9(1):e52-e60.
4. Bartsch SM, Hotez PJ, Asti L, Zapf KM, Bottazzi ME, Diemert DJ, et al. The global economic and health burden of human hookworm infection. PLoS neglected tropical diseases. 2016;10(9):e0004922.
5. Pullan RL, Smith JL, Jasrasaria R, Brooker SJ. Global numbers of infection and disease burden of soil transmitted helminth infections in 2010. Parasites & Vectors. 2014;7(1):1-19.
6. Hotez PJ, Kamath A. Neglected tropical diseases in sub-Saharan Africa: review of their prevalence, distribution, and disease burden. PLoS Neglected Tropical Diseases. 2009;3(8):e412.
7. Assefa G, Alemu M, Ayehu A. High Prevalence of Hookworm Species and Associated Factors among Soil-Transmitted Helminth-Infected Household Contacts in Burie Zuria District, Northwest Ethiopia: A Community-Based Cross-Sectional Study. BioMed Research International. 2023.

8. Anegagrie, M., Lanfri, S., Aramendia, A. A., Scavuzzo, C. M., Herrador, Z., Benito, A., et al. (2021). Environmental characteristics around the household and their association with hookworm infection in rural communities from Bahir Dar, Amhara Region, Ethiopia. PLoS Neglected Tropical Diseases, 15, e00094669.

9. Leta GT, Mekete K, Wuletaw Y, Gebretsadik A, Sime H, Mekasha S, et al. National mapping of soil-transmitted helminth and schistosome infections in Ethiopia. Parasites & Vectors. 2020;13(1):1-13.

10. Alelign T, Degarege A, Erko B. Soil-transmitted helminth infections and associated risk factors among schoolchildren in Durbete Town, Northwestern Ethiopia. Journal of Parasitology Research. 2015;2015.

11. Nute AW, Endeshaw T, Stewart AE, Sata E, Bayissasse B, Zerihun M, *et al*. Prevalence of soil-transmitted helminths and Schistosoma mansoni among a population-based sample of school-age children in Amhara region, Ethiopia. Parasites & Vectors. 2018 a;11(1):1-9.

12. Campbell SJ, Nery SV, McCarthy JS, Gray DJ, Magalhães RJS, Clements AC. A critical appraisal of control strategies for soil-transmitted helminths. Trends in Parasitology. 2016;32(2):97-107.

13. Mationg MLS, Williams GM, Tallo VL, Olveda RM, Aung E, Alday P, et al. Determining the impact of a school-based health education package for prevention of intestinal worm infections in the Philippines: protocol for a cluster randomized intervention trial. JMIR Research Protocols. 2020;9(6):e18419.

14. Isah AUJ, Ekwunife OI, Ejie IL, Mandrik O. Effects of nutritional supplements on the re-infection rate of soil-transmitted helminths in school-age children: A systematic review and meta-analysis. PLoS One. 2020;15(8):e0237112.

15. Quinnell R, Slater A, Tighe P, Walsh E, Keymer A, Pritchard D. Reinfection with hookworm after chemotherapy in Papua New Guinea. Parasitology. 1993;106(4):379-85.
16. Yap P, Du Z-W, Wu F-W, Jiang J-Y, Chen R, Zhou X-N, et al. Rapid re-infection with soil-transmitted helminths after triple-dose albendazole treatment of school-aged children in Yunnan, People's Republic of China. The American Journal of Tropical Medicine and Hygiene. 2013;89(1):23.
17. Dunn JC, Bettis AA, Wyine NY, Lwin AMM, Tun A, Maung NS, et al. Soil-transmitted helminth reinfection four and six months after mass drug administration: results from the delta region of Myanmar. PLoS Neglected Tropical Diseases. 2019;13(2):e0006591.
18. WHO. 2030 targets for soil-transmitted helminthiases control programmes. 2020.
19. Tello R, Terashima A, Marcos LA, Machicado J, Canales M, Gotuzzo E. Highly effective and inexpensive parasitological technique for diagnosis of intestinal parasites in developing countries: spontaneous sedimentation technique in tube. International Journal of Infectious Diseases. 2012;16(6):e414-e6.
20. WHO. Bench aids for the diagnosis of intestinal parasites: World Health Organization; 2019.
21. Vercruysse J, Behnke JM, Albonico M, Ame SM, Angebault C, Bethony JM, et al. Assessment of the anthelmintic efficacy of albendazole in school children in seven countries where soil-transmitted helminths are endemic. PLoS Neglected Tropical Diseases. 2011;5(3):e948.
22. Federal ministry of health (FmoH). The Third National Neglected Tropical Diseases Strategic Plan 2021-2025. 2021.
23. Rahmi S, Anwar C, Hasyim H, Amin R, Ghiffari A. The Correlation of No Footwear Use and Soil Helminth Incidence among Elementary School Children in Musi Rawas, South Sumatera, Indonesia. Bioscientia Medicina: Journal of Biomedicine and Translational Research. 2021;5(12):1217-22.

24. Zeleke AJ, Addisu A, Derso A, Tegegne Y, Birhanie M, Sisay T, et al. Evaluation of hookworm diagnosis techniques from patients in Debre Elias and Sanja Districts of the Amhara Region, Ethiopia. Journal of Parasitology Research. 2021;2021.
25. Goshu A, Alemu G, Ayehu A. Prevalence and Intensity of Soil-Transmitted Helminths and Associated Factors among Adolescents and Adults in Bibugn Woreda, Northwest Ethiopia: A Community-Based Cross-Sectional Study. Journal of Tropical Medicine. 2021;2021.
26. Speich B, Moser W, Ali SM, Ame SM, Albonico M, Hattendorf J, et al. Efficacy and reinfection with soil-transmitted helminths 18-weeks post-treatment with albendazole-ivermectin, albendazole-mebendazole, albendazole-oxantel pamoate and mebendazole. Parasites & Vectors. 2016;9(1):1-10.
27. Aschale Y, Abebaw A, Atnaf A, Mengist A, Kassie B, Yihunie W. Hookworm re-infection rate and efficacy of single-dose albendazole among pregnant women in Debre Elias District, Northwest Ethiopia: A single-arm trial. Tropical Doctor. 2022:00494755221080593.
28. Zerdo Z, Yohanes T, Tariku B. Soil-transmitted helminth reinfection and associated risk factors among school-age children in Chencha District, southern Ethiopia: a cross-sectional study. Journal of Parasitology Research. 2016.
29. Al-Mekhlafi MH, Surin J, Atiya A, Ariffin W, Mahdy AM, Abdullah HC. Pattern and predictors of soil-transmitted helminth reinfection among aboriginal schoolchildren in rural Peninsular Malaysia. Acta Tropica. 2008;107(2):200-4.
30. Jia TW, Melville S, Utzinger J, King CH, Zhou XN. Soil-transmitted helminth reinfection after drug treatment: a systematic review and meta-analysis. PLoS Negl Trop Dis. 2012;6(5):e1621.
31. Zeleke AJ, Bayih AG, Afework S, Gilleard JS. Treatment efficacy and re-infection rates of soil-transmitted helminths following mebendazole treatment in schoolchildren, Northwest Ethiopia. Tropical Medicine and Health. 2020;48(1):1-6.

32. Okoyo C, Campbell SJ, Williams K, Simiyu E, Owaga C, Mwandawiro C. Prevalence, intensity and associated risk factors of soil-transmitted helminth and schistosome infections in Kenya: impact assessment after five rounds of mass drug administration in Kenya. PLoS Neglected Tropical Diseases. 2020;14(10):e0008604.
33. Tiruneh T, Geshere G, Ketema T. Prevalence and determinants of soil-transmitted helminthic infections among school children at goro primary school, South West Shewa, Ethiopia. International Journal of Pediatrics. 2020.
34. Tolera A, Dufera M. The prevalence of soil-transmitted helminths and associated risk factors among school children at Sekela Primary School, Western Ethiopia. Journal of Parasitology Research. 2020;2020.
35. Nery SV, Clarke NE, Richardson A, Traub R, McCarthy JS, Gray DJ, et al. Risk factors for infection with soil-transmitted helminths during an integrated community level water, sanitation, and hygiene and deworming intervention in Timor-Leste. International Journal for Parasitology. 2019;49(5):389-96.

Click here to access/download
Supporting Information
Ethical paper.pdf
