# Supplementary material for: Pattern and predictor of hookworm re-infection among schoolchildren in three districts of Amhara Region, northwest Ethiopia
Source: PLoS One. 2024 May 28;19(5):e0303502. doi: 10.1371/journal.pone.0303502 (PMC11132481; doi:10.1371/journal.pone.0303502)
Supplement: Attachment — Submitted filename: Authors response to Reviewers comments.docx [file pone.0303502.s002.docx]

**Authors ‘ response to reviewers’ comments**

*First, we would like acknowledge the editor and the reviewers for your constructive comments and questions. We have learned much from your comments and we have tried to respond to your comments in the manuscript.*

PONE-D-23-41533

Pattern and predictor of hookworm re-infection among schoolchildren in three districts of

Journal Requirements:

1. When submitting your revision, we need you to address these additional requirements.

Please ensure that your manuscript meets PLOS ONE's style requirements, including those for file naming. The PLOS ONE style templates can be found at 
<https://journals.plos.org/plosone/s/file?id=wjVg/PLOSOne_formatting_sample_main_body.pdf> and 
<https://journals.plos.org/plosone/s/file?id=ba62/PLOSOne_formatting_sample_title_authors_affiliations.pdf>

**Authors’ response:** The PLOS ONE style has been maintained**.**

2. We note that another paper by the same author group and with the title [“Performance Evaluation of Three Diagnostic Methods for Soil-Transmitted Helminth Infections among Schoolchildren in Amhara Region, Northwest Ethiopia] was recently published. It has been noted that the two submissions may have some overlap with regard to the population cohort. We ask that you please clarify why you feel the two reports should be considered as separate articles in your cover letter. In your manuscript, please ensure you cite, discuss, and acknowledge overlap with the related work, and provide adequate justification (in the Introduction) for the new submission in light of the related published work.

**Authors’ response:** I appreciate the editors concern. In our project, we had two main objectives. The first objective was to evaluate the performance of diagnostic methods against STHs in the baseline data. The second objective was to identify the patterns and predictor factors of hookworm infection after treatment has been given. Although the two articles are conducted in the same population, the have different objectives. In any case I cited the first title in this manuscript (line 267-68)

3. We note that your Data Availability Statement is currently as follows: All relevant data are within the manuscript and its Supporting Information files.

Please confirm at this time whether or not your submission contains all raw data required to replicate the results of your study. Authors must share the “minimal data set” for their submission. PLOS defines the minimal data set to consist of the data required to replicate all study findings reported in the article, as well as related metadata and methods (<https://journals.plos.org/plosone/s/data-availability#loc-minimal-data-set-definition>).

For example, authors should submit the following data:

- The values behind the means, standard deviations and other measures reported;
- The values used to build graphs;

- The points extracted from images for analysis.

Authors do not need to submit their entire data set if only a portion of the data was used in the reported study.

If your submission does not contain these data, please either upload them as Supporting Information files or deposit them to a stable, public repository and provide us with the relevant URLs, DOIs, or accession numbers. For a list of recommended repositories, please see <https://journals.plos.org/plosone/s/recommended-repositories>.

If there are ethical or legal restrictions on sharing a de-identified data set, please explain them in detail (e.g., data contain potentially sensitive information, data are owned by a third-party organization, etc.) and who has imposed them (e.g., an ethics committee). Please also provide contact information for a data access committee, ethics committee, or other institutional body to which data requests may be sent. If data are owned by a third party, please indicate how others may request data access.

**Authors’ response:** “All relevant data are within the manuscript and its Supporting Information files has been included in the manuscript.”

4. When completing the data availability statement of the submission form, you indicated that you will make your data available on acceptance. We strongly recommend all authors decide on a data sharing plan before acceptance, as the process can be lengthy and hold up publication timelines. Please note that, though access restrictions are acceptable now, your entire data will need to be made freely accessible if your manuscript is accepted for publication. This policy applies to all data except where public deposition would breach compliance with the protocol approved by your research ethics board. If you are unable to adhere to our open data policy, please kindly revise your statement to explain your reasoning and we will seek the editor's input on an exemption. Please be assured that, once you have provided your new statement, the assessment of your exemption will not hold up the peer review process.

**Authors’ response:** Our entire data will need to be made freely accessible if our manuscript is accepted for publication.

5. PLOS requires an ORCID iD for the corresponding author in Editorial Manager on papers submitted after December 6th, 2016. Please ensure that you have an ORCID iD and that it is validated in Editorial Manager. To do this, go to ‘Update my Information’ (in the upper left-hand corner of the main menu), and click on the Fetch/Validate link next to the ORCID field. This will take you to the ORCID site and allow you to create a new iD or authenticate a pre-existing iD in Editorial Manager. Please see the following video for instructions on linking an ORCID iD to your Editorial Manager account: <https://www.youtube.com/watch?v=_xcclfuvtxQ>

**Authors’ response:** I have already had ORCID iD

6. Please include captions for your Supporting Information files at the end of your manuscript, and update any in-text citations to match accordingly. Please see our Supporting Information guidelines for more information: <http://journals.plos.org/plosone/s/supporting-information>. 

**Authors’ response:** Okay!

Reviewers' comments:

**Comments to the Author**

1. Is the manuscript technically sound, and do the data support the conclusions?

The manuscript must describe a technically sound piece of scientific research with data that supports the conclusions. Experiments must have been conducted rigorously, with appropriate controls, replication, and sample sizes. The conclusions must be drawn appropriately based on the data presented.

Reviewer #1: Partly

Reviewer #2: Yes

Reviewer #3: Yes

**Authors’ response:** The conclusions are drown from the main findings of the study.

2. Has the statistical analysis been performed appropriately and rigorously?

Reviewer #1: Yes

Reviewer #2: I Don't Know

Reviewer #3: I Don't Know

**Authors’ response:** The main findings have been analyzed properly.

3. Have the authors made all data underlying the findings in their manuscript fully available?

The [PLOS Data policy](http://www.plosone.org/static/policies.action#sharing) requires authors to make all data underlying the findings described in their manuscript fully available without restriction, with rare exception (please refer to the Data Availability Statement in the manuscript PDF file). The data should be provided as part of the manuscript or its supporting information, or deposited to a public repository. For example, in addition to summary statistics, the data points behind means, medians and variance measures should be available. If there are restrictions on publicly sharing data—e.g. participant privacy or use of data from a third party—those must be specified.

Reviewer #1: No

Reviewer #2: Yes

Reviewer #3: Yes

**Authors’ response:** All data which supports the findings have been available in the manuscript.

4. Is the manuscript presented in an intelligible fashion and written in standard English?

PLOS ONE does not copyedit accepted manuscripts, so the language in submitted articles must be clear, correct, and unambiguous. Any typographical or grammatical errors should be corrected at revision, so please note any specific errors here.

Reviewer #1: Yes

Reviewer #2: Yes

Reviewer #3: No

**Authors’ response:** The language is clear and correct and has been improved based on the given comments.

5. Review Comments to the Author

Please use the space provided to explain your answers to the questions above. You may also include additional comments for the author, including concerns about dual publication, research ethics, or publication ethics. (Please upload your review as an attachment if it exceeds 20,000 characters)

Reviewer #1: The study by Belay et. al., titled "Patterns of Hookworm Reinfection Rates and Predictors among Schoolchildren in Northwest Ethiopia" aims to investigate the re-infection rates and predictors of hookworm among schoolchildren in northwest Ethiopia. While the study addresses an important public health issue, there are a few queries related to its design and methodology to assess the robustness of the findings and the implications for future research and interventions.

**Authors’ response:** Thank you for comment.

**Major Queries:**

External Validity: To what extent can the findings of the study be generalized to other populations of schoolchildren in Ethiopia or similar settings with high hookworm prevalence? What measures were taken to ensure the representativeness of the study sample?

**Authors’ response:** I appreciate the reviewer’s concern. The current study was conducted in three districts. One school was selected in each district. To insure the representativeness of the study, districts, schools and schoolchildren was selected randomly. In addition, to make it representative, two schools were selected from the rural and one from the city. So, the data obtained in this study can be generalized to other populations of schoolchildren in Ethiopia or similar settings with high hookworm prevalence.

Methodological Rigor: How were potential sources of bias, such as selection bias or measurement bias, addressed during the study design and data collection phases? Were steps taken to minimize the risk of misclassification or misreporting of predictor variables?

**Authors’ response:** To address the selection bias during checking the patterns of re-infection rate, first we selected those schoolchildren who were positive in the baseline data and treated with albendazole and became negative at day 14^th^. All schoolchildren negative by the three diagnostic methods were selected to avoid selection bias using different diagnostic methods. Similar laboratory testing was also done in the 4^th^ and 6^th^ months. To mimimize the bias, misclassification and misreporting were check at the 14^th^ day of post treatment, 4^th^ and 6^th^ months of checking.

Longitudinal Dynamics: Given the short follow-up period of six months, how do the observed re-infection rates and predictors align with longer-term patterns of hookworm transmission and control? What implications do these findings have for the sustainability of intervention strategies over time?

**Authors’ response:** I appreciate the reviewer’s concern regarding the short follow-up period of six months. I understand the long term follow-up overweight’s the short follow-up period. Although our study was followed for six months due to limited budget, the aliment of predictors in the 4^th^ and 6^th^ month was interesting. In hookworm endemic areas, and people are practicing bare footed, the re-infection rate occurs in very short period. Therefore, the intervention strategy should be strongly advocated using integrated approach (WASH, MDA and Health education). Despite the study conducted in short period of time, it advances the understanding of hookworm transmission dynamics and improves public health outcomes in affected communities, especially in endemic areas.

Comprehensive Assessment: Are there additional predictor variables or potential confounders that were not included in the analysis but may significantly influence hookworm re-infection rates among schoolchildren? How could future research efforts broaden the scope of predictor assessment to capture a more comprehensive range of factors?

**Authors’ response:** I appreciate the reviewers concern regarding additional variables. There are some environmental factors such as soil type, temperature, vegetation, rain fall and wet environment which might have an influence on hookworm re-infection rates. These factors were not assessed in this study due to limited budget. Further comprehensive assessment of predictor variable including personal, habits, and environmental factors should be conducted to address the re-infection rates of hookworm re-infection rates.

Intervention Strategies: Based on the identified predictors of hookworm re-infection, what targeted intervention strategies or public health interventions could be implemented to reduce transmission rates and improve outcomes among schoolchildren in northwest Ethiopia? How can these strategies be tailored to address the specific needs and challenges of the local community? Some discussion about them should be included in the manuscript.

**Authors’ response**: I appreciate the reviewers regarding targeted interventions. To reduce transmission rates and decrease the outcomes of hookworm re-infection rate among schoolchildren, emphasis should be given for prevention using an integrated approach, which includes proper implementation of water sanitation and hygiene, especially in the rural settings, periodic deworming using albendazole and community based health education (how hookworm infection is transmitted and prevented?) should be conducted in northwest Ethiopia. Based on our results, proper latrine utilization and shoes wearing during outdoor activities like farming and irrigation should be targeted to intervene hookworm re-infection.

Minor point: Line 124 -the word principal investigator is misspelled as "investigettor".

**Authors’ response**: Correction has been done (line 131).

Overall, while the study contributes valuable insights into hookworm re-infection rates and predictors among schoolchildren in northwest Ethiopia, it also advances our understanding of hookworm transmission dynamics and improves public health outcomes in affected communities.

**Authors’ response**: Thank you very much!

Reviewer #2: The authors performed a study regarding hookworm re-infection among schoolchildren in northwest Ethiopia. This is an interesting study, but it needs some revisions-clarification:
1) Please use the update data and references for lines 38-40.

**Authors’ response:** Updated reference have been used (line 38-40).

2) Did you consider nutritional habits, such as eating raw vegetables, etc, for the selected cases.

**Authors’ response:** Since faeco-oral transmission of hookworm is rare, we could not considered nutritional habits.

**Reviewer#3: PONE-D-23-41533**
Pattern and predictor of hookworm re-infection among schoolchildren in three districts of Amhara Region, northwest Ethiopia

PLOS ONE

**Reviewer’s Comments**

The manuscript describes the findings of baseline faecal surveys conducted among school children in three districts of Amhara region, northwest Ethiopia, and follow up faecal surveys conducted among hookworm (HW) positive subjects at 4th and 6th month after deworming with albendazole with the objective of determining the reinfection rates of hookworm infection and predictive factors associated with HW reinfection. Although the methodology and results satisfy the study objectives there are many shortcomings in the manuscript preparation compromising the scientific validity of the manuscript. My queries and comments are herewith listed.
1. The Introduction describes the HW infection status as “high prevalence” in rural Amhara region. Is there any information in the literature regarding the species distribution of HWs causing infection in the region? Which may be useful to include in the Introduction.

**Authors’ response**: As far as my knowledge is concerned, there is no previous hookworm species data in the region.

2. Likewise, the endemicity of other geohelminth infections in the region if known should be included in the introduction.

**Authors’ response**: The endemicity of other geohelminth infection is not well documented and known in the region.

3. Line 41-42, “despite control measures implemented…..” Please include a brief description of the control measures implemented under Introduction or discussion.

**Authors’ response**: Revision has been done in the introduction section. (line 42-43) and in the discussion (line 273-74)

4. Under Methods, a brief description of the areas studied need to be included such as climatic conditions including vegetation, water supply, sanitary facilities, rural or urban (which has been mentioned) and routine deworming programs if any etc.

**Authors’ response**: Revision has been done in the method section (line 67-71).

5. Brief description of sample size calculation to be included under methodology.

**Authors’ response**: The sample size calculation has been included in the study (line 72-74).

6. Under results, the baseline faecal surveys report only HW infections which is surprising in the presence of widespread faecal contamination of the environment as described in this manuscript. Probable explanations need to be included in the discussion as to why there is only a high prevalence of HW infections and absence of other geohelminth species.

**Authors’ response**: Explanation about the high prevalence of hookworm than other geohelminths has been discussed in discussion part (line 294-96).

7. There were multiple spelling and grammatical errors, some of which I have highlighted in the edited manuscript. The manuscript could be improved with English language editing.

**Authors’ response:** Correction has been done including highlighted in the edited manuscript.
